# How Hyperarousal and Sleep Reactivity Are Represented in Different Adult Age Groups: Results from a Large Cohort Study on Insomnia

**DOI:** 10.3390/brainsci7040041

**Published:** 2017-04-14

**Authors:** Ellemarije Altena, Ivy Y. Chen, Yannick Daviaux, Hans Ivers, Pierre Philip, Charles M. Morin

**Affiliations:** 1 Sommeil, Addiction et Neuropsychiatrie, Univ. Bordeaux, USR 3413, F-33000 Bordeaux, France; yannick.daviaux@u-bordeaux.fr (Y.D.); pr.philip@free.fr (P.P.); 2 Sommeil, Addiction et Neuropsychiatrie, CNRS, USR 3413, F-33000 Bordeaux, France; 3 École de psychologie, Université Laval, Québec City, QC G1V 0A6, Canada; ivy.y.m.chen@gmail.com (I.Y.C.); Hans.Ivers@psy.ulaval.ca (H.I.); cmorin@psy.ulaval.ca (C.M.M.); 4 Centre d’Étude des Troubles du Sommeil, Centre de Recherche de l’Institut Universitaire en Santé Mentale de Québec, Quebec City, QC G1J 2G3, Canada

**Keywords:** insomnia, sleep reactivity, arousal, age

## Abstract

Hyperarousal is a 24-h state of elevated cognitive and physiological activation, and is a core feature of insomnia. The extent to which sleep quality is affected by stressful events—so-called sleep reactivity—is a vulnerability factor for developing insomnia. Given the increasing prevalence of insomnia with age, we aimed to investigate how hyperarousal and sleep reactivity were related to insomnia severity in different adult age groups. Data were derived from a large cohort study investigating the natural history of insomnia in a population-based sample (*n* = 1693). Baseline data of the Arousal Predisposition Scale (APS) and Ford Insomnia Response to Stress Test (FIRST) were examined across age and sleep/insomnia subgroups: 25–35 (*n* = 448), 35–45 (*n* = 528), and 45–55 year olds (*n* = 717); good sleepers (*n* = 931), individuals with insomnia symptoms (*n* = 450), and individuals with an insomnia syndrome (*n* = 312). Results from factorial analyses of variance (ANOVA) showed that APS scores decreased with increasing age, but increased with more severe sleep problems. FIRST scores were not significantly different across age groups, but showed the same strong increase as a function of sleep problem severity. The findings indicate that though arousal predisposition and sleep reactivity increase with more severe sleep problems, only arousal decreases with age. How arousing events affect an individual during daytime thus decreases with age, but how this arousal disrupts sleep is equivalent across different adult age groups. The main implication of these findings is that treatment of insomnia could be adapted for different age groups and take into consideration vulnerability factors such as hyperarousal and stress reactivity.

## 1. Introduction

Insomnia is a prevalent condition in all ages, but particularly in middle-aged and older adults [1]. A core feature of insomnia is hyperarousal—a 24-h state of elevated physiological and cognitive activation [2]. Hyperarousal is well recognized as a risk factor for developing insomnia [3]. A measure particularly sensitive for measuring hyperarousal is the Arousal Predisposition Scale (APS) [4], which has provided insight into the intermediating role that arousal plays between stress and sleep [5]. Scores on this questionnaire have been shown to be higher in insomnia syndrome incident cases compared to good sleepers [3].

The extent to which sleep is affected by stressful events—so-called sleep reactivity—is another factor that has been hypothesized to increase vulnerability for developing insomnia. The degree of sleep reactivity in an individual can be assessed by the Ford Insomnia Response to Stress Test (FIRST) [6]. Higher sleep reactivity has been associated with an increased risk of incident insomnia syndrome one year later among good sleepers [7] and strongly related to arousal and emotion coping in insomnia [8].

Older adults may be less affected by stress through habituation and improved coping strategies, which are important factors driving sleep quality [5,9,10]. Few studies investigated age differences in immediate and delayed stress reactions, and those who do mostly focus on older age groups (over 65 years) [11,12]. However, a large study (*n* = 190) applying ecological momentary assessment (EMA) five times a day in 20- to 81-year-olds to measure positive and negative affect has shown that older adults, as opposed to younger adults, are less affected by stressors. However, no age differences were observed in the effects of the stressor three to six hours after exposure [13]. Studies that investigate how age affects the stress–arousal–sleep relationship are lacking as of yet.

Sleep becomes more disrupted with age, and part of those changes is explained by the increased incidence of health problems with aging. In addition, there are other developmental changes that may impact sleep quality with aging, including neurocognitive and hormonal changes, behavioural/sleep scheduling factors, and reduced amplitude of circadian rhythms [14]. These factors may attenuate the role of arousal as a factor accounting for sleep disturbances. Our goal was to examine the impact of hyperarousal and sleep reactivity as insomnia vulnerability factors across age groups, while at the same time minimizing the potential confound of these variables. As such, we can investigate the importance of hyperarousal and sleep reactivity as vulnerability factors for insomnia and its changes within older adult age.

In the current study, we thus investigate how hyperarousal and sleep reactivity are related to insomnia severity in a large cross-sectional study comparing different adult age groups from 25 to 55 years old. We first hypothesize that both factors are strongly related to existing sleep problems, independent of age. We expect that hyperarousal decreases with age, but sleep reactivity remains equal independent of sleep problems. Findings can give insight into factors playing a role in vulnerability to insomnia in different age groups, which may facilitate personalized treatment adapted to different age groups, aimed not just at treating sleep problems, but also at normalizing arousal and sleep reactivity.

## 2. Materials and Methods

### 2.1. Participants

Data were derived from a large cohort study investigating the natural history of insomnia in a population-based sample. Participants were chosen using a stratified probabilistic selection procedure based on the last Canadian census, combined with a random digit selection method and the Kish method to identify which household member was interviewed [15]. Inclusion criteria for the telephone interview were to be over 18 years of age and to speak French or English [16]. A total sample size of 3911 was acquired initially.

From this cohort, we selected baseline data for the age group between 25 and 55 years of age (*n* = 1693) in order to reduce the impact of potential confounding factors. Specifically, individuals in this age range may have a more regular lifestyle and day-and-night rhythm than in later life phases, and other age- and health-related problems disrupting sleep quality such as menopause or neurodegenerative diseases are not yet prevalent.

### 2.2. Measures

While DSM criteria are leading in the diagnosis of insomnia, the Insomnia Severity Index (ISI) [17] is a well-accepted measure that assesses severity of nocturnal symptoms and daytime impairments [17,18]. It is a seven-item self-administered questionnaire asking about severity of sleep difficulties (initial, middle, and late insomnia), satisfaction about actual sleep quality, perceived daytime impairments, as well as thoughts and worries about sleep. Responses to each item range from 0 (e.g., no difficulty) to 4 (e.g., very difficult). The ISI has adequate psychometric properties and is sensitive to measure treatment outcome. The version applied here—the French-Canadian version of the ISI—has good internal consistency, test–retest reliability (Cronbach’s alpha of 0.74), and convergent validity (r = 65 when comparing with sleep diary) [17]. Arousal was measured using the Arousal Predisposition Scale [4], which assesses through 12 items whether the respondent considers her/himself a stressful and emotionally reactive person. It is a self-report scale that inquires about typical behaviours and emotional responses to daytime events, either with or without an external trigger. Each item is scored on a scale from 1 (never) to 5 (always). The APS has good predictive validity and internal consistency (Cronbach’s alpha of 0.83) [19]. Sleep reactivity was measured using the Ford Insomnia Response to Stress Test [6], which contains nine items assessing whether past and future stressful events are likely to affect sleep quality. It is a self-report scale that assesses the likelihood of having trouble sleeping either after a stressful or arousing event during the previous day or when anticipating this event for the following day. For each item, scores run from 0 (not likely) to 4 (very likely). The FIRST is a reliable measure with high internal consistency (Cronbach’s alpha of 0.83) and good test–retest reliability (t-rr coefficient = 92) [6].

### 2.3. Analyses

Of the 1693 participants between 25 and 55 years of age, we first analysed how vulnerability to insomnia actually affected insomnia severity scores by comparing groups with high and low scores on the APS and FIRST. Groups were created based on a median split for both arousal and sleep reactivity scores, with low vulnerability defined as a score lower than median and high vulnerability as a score equal to or higher than the median. We first analysed how high and low arousal and sleep reactivity were related to insomnia severity separately. Based on the results, four groups were then created: a high insomnia vulnerability group (high vulnerability scores on both arousal and sleep reactivity scales), a low vulnerability group (low vulnerability scores on both arousal and sleep reactivity scales), and medium vulnerability groups (a high score on one and a low score on the other scale). For each of these groups, we calculated the mean and standard deviation (SD) of ISI scores. By performing the analyses in these stages, we could determine whether it was one of these vulnerability factors alone that would drive insomnia severity scores, or whether these factors combined had a much stronger effect on insomnia severity scores than either of these factors alone.

Next, we investigated the age effect on sleep reactivity and arousal. Baseline data were examined across three different age groups: 25–35 year olds, 35–45 year olds, and 45–55 year olds. In parallel and across age, the group was divided into three different sleep groups based on a previously described algorithm [20]: good sleepers, those with insomnia symptoms, and those with insomnia syndrome. The algorithm considers criteria of the Diagnostic and Statistical Manual of Mental Disorders, 4th Edition (DSM-IV [21]) and International Classification of Diseases, 10th Edition [22]) as well as sleep-promoting medication use and scores on the ISI and Pittsburgh Sleep Quality Index [23]. Responses from the Insomnia Severity Index [18] the Pittsburgh Sleep Quality Index [23], and from questions about the use of sleep medication were used to evaluate the presence or absence of each criterion. Participants in the group with an insomnia syndrome met all diagnostic criteria for insomnia. They were dissatisfied or very dissatisfied (score of 3 or 4 on a scale of 0–4) with their sleep patterns and had symptoms of initial, middle, or late insomnia at least three nights per week for at least 1 month. Substantial distress or daytime impairment related to sleep difficulties was also reported by those individuals (score of 3 or 4 on a scale of 0–4). Participants were also classified in the insomnia syndrome group if they used prescribed sleep-promoting medication at least three nights per week. Although not a formal criterion for insomnia diagnosis, use of sleep medication may mask the underlying symptoms. Participants classified in the group with insomnia symptoms reported initial, middle, or late insomnia at least three nights per week without fulfilling all diagnostic criteria for insomnia syndrome (i.e., they could report being satisfied with their sleep, not report distress or daytime consequences, or not meet the criterion of symptoms for at least 1 month required for a diagnosis of insomnia). This group also included individuals dissatisfied with their sleep but without symptoms of initial, middle, or late insomnia. Participants using prescribed sleep medication fewer than three nights per week or over-the-counter medication for sleep at least one night per week were classified in this group. Participants in the good sleepers group were satisfied with their sleep (i.e., score of 0–2 on a scale of 0–4) did not report symptoms of insomnia, and did not use prescribed or over-the-counter medication to promote sleep.

The group with insomnia syndrome fulfilled all criteria of insomnia diagnosis (*n* = 312), those with insomnia symptoms showed some but not all symptoms of insomnia (*n* = 450), and the group of good sleepers did not complain about their sleep nor did they take any sleep medication (*n* = 931). A multi-factorial ANOVA was conducted to compare the main effects of age group and sleep group and the interaction effect of both on APS and FIRST scores.

## 3. Results

### 3.1. Insomnia Severity in High and Low Vulnerability Groups

Medians for both arousal (APS, median = 30) and sleep reactivity (FIRST, median = 22) were first calculated. Defining high and low arousal and sleep reactivity groups based on these values is in line with previously published high arousal and high sleep reactivity values, and with those defined in the original test definitions [4,6]. Participants with high arousal scores (APS, *n* = 893) had significantly higher scores on the insomnia severity index (ISI: Mean = 10.29, SD = 5.81) than participants with low arousal scores (*n* = 800, Mean = 6.72, SD = 5.12) (*F*_1, 1691_ = 175.138, *p* < 0.001). Likewise, participants with high reactivity scores (*n* = 919) reported significantly higher insomnia severity (Mean = 10.57, SD = 5.68) than those with low sleep reactivity (*n* = 774; Mean = 6.27, SD = 5.05) (*F*_1, 1691_ = 267.523, *p* < 0.001).

These data supported our creation of a second level division in vulnerability groups, combining arousal and sleep reactivity scores as described in the methods. We created a group with high scores on both arousal and sleep reactivity (high vulnerability, *n* = 630). Medium vulnerability was defined as having high scores on one but low score on the other questionnaire, and so consisted of two groups: one group with high arousal and low sleep reactivity scores (*n* = 263), and one group with high sleep reactivity and low arousal scores (*n* = 289). Lastly, a low vulnerability group was created with low scores on both arousal and sleep reactivity measures (*n* = 511).

**Figure 1:**
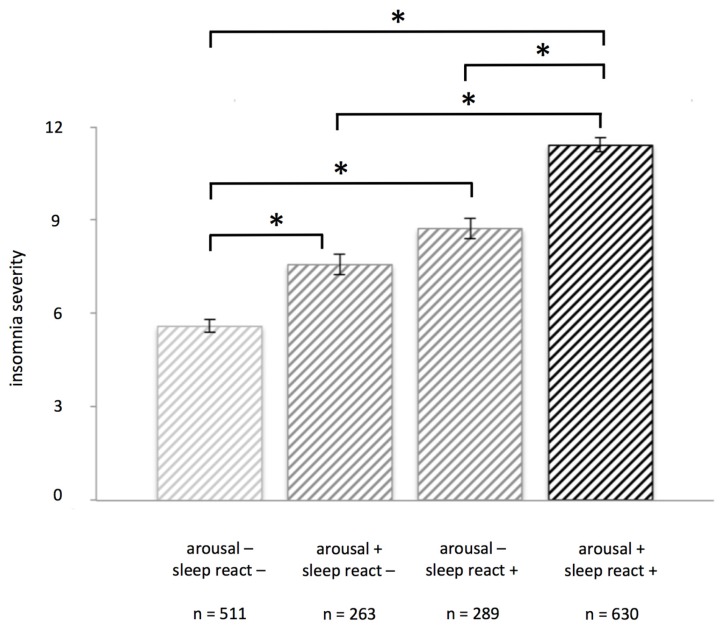
Insomnia severity scores vary depending on low, medium, or high insomnia vulnerability. Those with low vulnerability for insomnia (scoring low on both arousal and sleep reactivity) have significantly lower scores on insomnia severity than those with medium (scoring high on only one scale) or high vulnerability (scoring high on both arousal and sleep reactivity). Groups were defined based on a medium split of arousal scores (arousal predisposition scale, APS) and sleep reactivity scores (Ford Insomnia Response to Stress Test, FIRST); error bars represent standard error of the mean. Asterisks (*) indicate a significance level of *p* < 0.001. See text for details.

There was a significant main effect of group on ISI scores (*F*_3, 1689_ = 118.37, *p* < 0.001). Post hoc tests showed that most groups were significantly different from each other: the high vulnerability group reported more severe insomnia (Mean = 11.42, SD = 5.60) than the medium vulnerability-SR group (Mean = 8.73, SD = 5.40, *p* < 0.001, d = 0.49), the medium vulnerability-AR group (Mean = 7.57, SD = 5.40, *p* < 0.001, d = 0.70), and the low vulnerability group (Mean = 5.59, SD = 4.72, *p* < 0.001, d = 1.13). The medium vulnerability-SR group and medium vulnerability-AR group each also reported significantly more severe insomnia than the low vulnerability group; (*p* < 0.001, d = 0.62) and (*p* < 0.001, d = 0.39), respectively. Only the medium vulnerability groups did not differ significantly from each other (*p* = 0.052, d = 0.21) (see Figure 1).

### 3.2. Hyperarousal and Sleep Reactivity as a Function of Age and Insomnia Symptoms

Next, we divided the initial total sample (*n* = 1693) into three age groups: 25–35 year olds (*n* = 448), 35–45 year olds (*n* = 528), and 45–55 year olds (*n* = 717). In parallel, three sleep/insomnia groups were formed based on our previously defined algorithm: a group of good sleepers (*n* = 931), those with symptoms of insomnia (*n* = 450), and those with insomnia syndrome (*n* = 312).

There was no significant age × sleep status interaction effect on either sleep reactivity (*F*_4, 1684_ = 0.27, *p* = 0.900) or on arousal (*F*_4, 1684_ = 0.59, *p* = 0.673). Results of the multivariate analysis of variance (MANOVA )for the sleep groups showed higher scores across each of the groups for both arousal (*F*_2, 1684_ = 68.340, *p* < 0.001) and sleep reactivity (*F*_2, 1684_ = 104.05, *p* < 0.001), confirming the results for insomnia vulnerability. Main effect of age showed that arousal scores are significantly lower with increasing age (*F*_2, 1684_ = 8.07, *p* < 0.001) but sleep reactivity scores are not (*F*_2, 1684_ = 0.45, *p* = 0.639).

**Figure 2:**
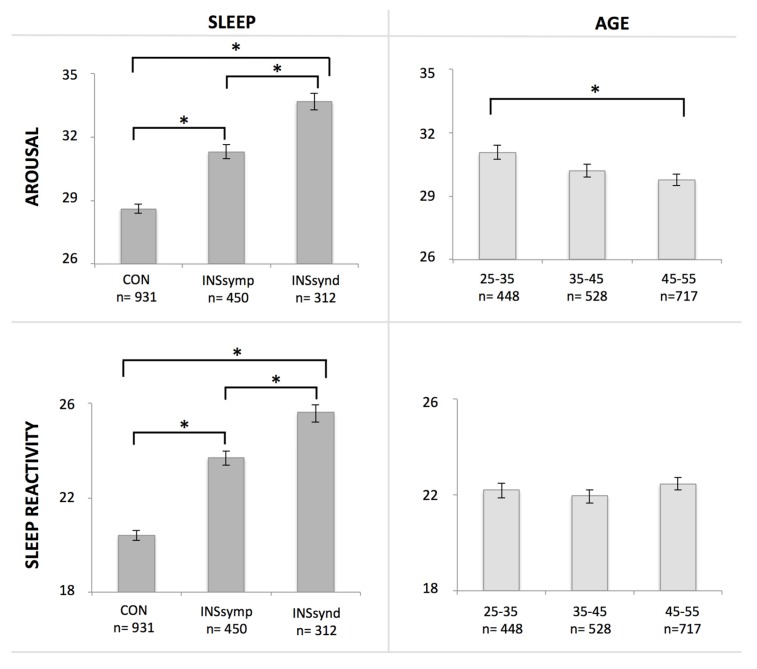
Arousal and sleep reactivity scores increase as a function of the presence and severity of sleep problems. Next to an overall significant main effect of group, post-hoc tests show significant differences between good sleepers (CON), those with insomnia symptoms (INS symp), and those with insomnia syndrome (INS synd) on both arousal and sleep reactivity scores. Arousal scores decrease with age: an overall main effect of age is supported by post-hoc test results of a significant difference between the younger (25–35 year olds) and older adult age group (45–55 year olds), although none of the other group comparisons are significantly different. Sleep reactivity scores are similar between the different age groups: there is no significant main effect nor are scores of any of the age groups significantly different from each other. Asterisks (*) indicate a significance level of *p* ≤ 0.005. See text for details.

Post-hoc comparisons using the Tukey’s honest significant difference (HSD) test revealed that arousal scores were significantly higher in the insomnia syndrome group (Mean = 33.69, SD = 7.14) compared to both the symptom group (Mean = 31.31, SD = 7.16, *p* < 0.001, d = −0.33) and to good sleepers (Mean = 28.60, SD = 6.61; *p* < 0.001, d = −0.74), while the insomnia symptom group also showed higher scores compared to the good sleepers (*p* < 0.001, d = −0.39). Significantly lower arousal scores were obtained among the 45–55 year olds (Mean = 29.78, SD = 7.27) compared to 25–35 year olds (Mean = 31.07, SD = 6.97; *p* = 0.005, d = 0.18), but not compared to 35–45 year olds (Mean = 30.22, SD = 7.07; *p* = 0.506, d = 0.06), nor did this last group differ significantly from the 25–35 year olds on arousal scores (*p* = 0.127, d = 0.12) (see Figure 2). For sleep reactivity, post-hoc comparisons using the Tukey HSD test revealed significantly higher scores in the insomnia syndrome group (Mean = 25.59, SD = 6.04) compared to both the symptom group (Mean = 23.72, SD = 6.06; *p* < 0.001, d = −0.31) and to good sleepers (Mean = 20.42, SD = 5.67; *p* < 0.001, d = −0.88), while the insomnia symptom group also showed higher scores compared to the good sleepers (*p* < 0.001, d = −0.56). Since a main effect of age was not found for sleep reactivity, post-hoc tests were not performed.

## 4. Discussion

The current study shows, in a large representative sample, that both arousal and sleep reactivity are strongly associated with sleep problems and that those with high scores on both measures particularly show high levels of insomnia severity. Furthermore, hyperarousal symptoms decrease as a function of age, but sleep reactivity remains similar across different age groups. How arousing events affect an individual during daytime decreases with age, but how stressful events disrupt sleep remains similar across different adult age groups.

Previous findings [24] have suggested that both arousal and sleep reactivity are relatively stable traits over shorter time periods (6 and 12 months follow-up). Though the current data are based on cross-sectional comparisons and thus limit conclusions about developmental changes over the lifespan, the data suggest that arousal but not sleep reactivity scores decrease in older adulthood. Findings are in line with a large study suggesting that the immediate stress response to an event decreases with age, but not delayed stress effects [13]. These delayed effects could—although not investigated—include effects on sleep.

Our findings have important implications for treatment adaptations for insomnia, which could be aimed at treating sleep problems but also at normalizing arousal and sleep reactivity. With stress reduction and improved emotion coping during daytime, it might be possible to reduce sleep-disrupting events during the night as well [14]. In fact, stress coping strategies are a mediating factor in the relation between stress exposure and insomnia development [25]. A new patient-based measure focusing not only on the likelihood but also on the actual frequency of sleep disrupting events might thus be warranted, in order to measure the effectiveness of such treatment. In fact, recent findings show that a reduction of insomnia symptoms through cognitive behavioural therapy is indeed associated with a reduction of sleep reactivity as measured by FIRST scores [26]. Sleep reactivity—as insomnia—is shown to be much more prevalent in women than in men, and is in turn associated with metacognitive beliefs about sleep in insomnia patients [27]. These findings emphasize the importance of reducing sleep reactivity through appropriate insomnia treatment. Longitudinal within-subject studies should further verify the hypothesis of trait and state within these different features of insomnia.

Despite insomnia being much more prevalent in older than in younger adults, the age-dependent decrease of arousal shown in the current study suggests that with increasing age, hyperarousal may play a less important role than other factors as a cause of insomnia. An interesting aim for future studies would be to map which factors are driving insomnia in older adults, improving the definition of insomnia phenotypes: reduced homeostatic drive, hormonal changes, changed diurnal rhythms due to changed lifestyles, and changes in sleep hygiene may all be factors underlying this higher insomnia prevalence in older adults instead of hyperarousal. The current study suggests that over a larger age span, the role that each of these factors play in the incidence and severity of insomnia varies with age for hyperarousal, but not for sleep reactivity.

The current study has a number of limitations: first of all, it is a cross-sectional and not a longitudinal study, so final conclusions on traits of hyperarousal and sleep reactivity await confirmation from such studies. Second, both insomnia diagnosis and reports of hyperarousal, sleep quality, and sleep reactivity were all assessed with subjective measures. This may also explain why the relative group size of those with insomnia syndrome is slightly higher than estimates from other prevalence studies, though numbers vary between studies [3,28,29]. Despite the limitations, the present findings give insight into factors playing a role in vulnerability to insomnia in different age groups, which may facilitate personalized treatment adapted to different age groups, aimed not just at treating sleep problems, but also at normalizing arousal and sleep reactivity, possibly preventing relapse of insomnia.

## 5. Conclusions 

Our analyses from a large representative cohort study show that both arousal predisposition and sleep reactivity increase with more severe sleep problems. Comparing different age groups between 25 and 55 years of age, we show that arousal predisposition, but not sleep reactivity scores decrease with increasing age. How arousing events affect an individual during daytime may thus decrease with age, but how arousal disrupts sleep is equivalent across different adult age groups. The findings implicate that treatment of insomnia should possibly be adapted for these age groups, taking into consideration vulnerability factors such as hyperarousal and sleep reactivity.
